# Comparison of Open Reduction and Internal Fixation With Minimally Invasive Plating in the Treatment of Distal Tibial Fractures: A Retrospective Study

**DOI:** 10.7759/cureus.23144

**Published:** 2022-03-14

**Authors:** Mehmet Akdemir, Ahmet Cemil Turan, Mehmet Aykut Türken, Çağdaş Biçen, Ali Ihsan Kilic

**Affiliations:** 1 Orthopedics, Izmir Ekol Hospital, Izmir, TUR; 2 Orthopedics and Traumatology, Private Clinic, Izmir, TUR; 3 Orthopedics and Traumatology, Izmir University of Economics Medicalpark Hospital, Izmir, TUR; 4 Orthopedics and Traumatology, Izmir University of Economics Medical Park Hospital, Izmir, TUR; 5 Orthopedics and Traumatology, Dokuz Eylul University, Izmir, TUR

**Keywords:** infection, skin necrosis, open reduction and internal fixation, minimal invasive plate, distal tibia fracture

## Abstract

Introduction

Treatment of distal tibial fractures may be problematic due to their close proximity to the ankle joint and poor skin coverage, resulting in skin problems, deep infection, and malunion. To address these problems, minimally invasive plating methods have been described. In this study, we aimed to compare the clinical findings, radiological findings, and complication rates of patients treated with open reduction or minimally invasive plating.

Methods

A total of 44 patients with distal tibial fractures with a mean follow-up period of 20.73 (12-50) months were included in this study retrospectively. The patients were divided into two groups, those who underwent open reduction and internal fixation and those treated with minimally invasive plates. The two groups were statistically compared in terms of radiological and clinical scores and complication rates (p=0.05). Comparative analysis was also performed for three fracture types in both groups.

Results

Twenty patients were treated with a minimally invasive approach and 24 patients were treated with the open reduction method. Age, gender, fracture type, and follow-up times were similar between the two groups (p>0.05). There was no statistically significant difference between postop American Orthopaedic Foot and Ankle Society Ankle-Hindfoot Score (AOFAS), anterior distal tibial angle (ADTA), or lateral distal tibial angle (LDTA) values between both groups. There was also no difference in union times or nonunion rates (p>0.05). There was no statistically significant difference in rates of superficial skin problems or deep infections between the two groups (p>0.05). In comparison regarding fracture types, patients with type C fractures seemed to have better outcomes with minimal invasive plating.

Conclusion

Minimally invasive plating is a good approach in the treatment of distal tibial fractures. The technique seems to be advisable, especially for patients with type C fractures. However, the rates of skin problems and deep infections are similar to those seen with the open reduction method.

## Introduction

Distal tibial fractures are special fractures. Bone damage may occur with axial loading and rotational and direct trauma [1]. The fracture line may extend to the ankle joint with the ankle joint being indirectly affected. This is a region in which false union (with shortness, angulation, and rotation) is less tolerated, and conservative treatment is therefore rarely preferred recently [2].

As an alternative to conservative treatment, open reduction and plating are nowadays preferred for lower rates of malunion and the anatomical ability of intra-articular restoration. However, the risk of skin necrosis and deep infection increases due to the relatively thin soft tissue covering this region, the wide dissection, and the opening of the joint [3].

The development of soft tissue problems has led to the need for alternative treatments. To overcome this problem, techniques that cause less damage to soft tissue have been introduced. These are; external fixation, intramedullary nailing, and minimally invasive plating. External fixation is less preferred for reasons such as low patient compliance, pin site infection, joint stiffness, and the possibility of re-fracture after fixator removal. However, it is preferred in cases of temporary use or infection until soft tissue healing [4]. Fracture reduction with an intramedullary nail can be very difficult. For this, polar screws and nails with more distal locks are required, an approach technically more difficult than the plating method. It is not recommended for patients with excessive intra-articular fragmentation [5].

In the minimally invasive method, small distal and proximal skin incisions and custom-made anatomical plates and locking screws are used without opening the fracture line. The goal is to prevent skin necrosis and deep infection with less dissection of soft tissues [6]. Joint restoration in intra-articular fractures can be performed with cannulated screws. Then, stabilization can be achieved by placing a minimally invasive plate. However, there are risks of insufficient fixation of the distal fracture fragment, nonunion due to insufficient compression at the fracture line, and irritation and infection due to the elevation of the plate [7]. As the fracture line and the joint cannot be seen directly, gradation and rotational deformity may occur in the joint [8].

In this study, we compare the radiological and clinical results of our patients treated with minimally invasive methods or open reduction plating for distal tibial fractures. We aim to determine whether there were differences in skin problems, union time, deep bone infection rate, and distal tibial angles between the two methods.

## Materials and methods

After obtaining the approval of the relevant ethics committee (Ege University Clinical Research Ethics Committee, date: 07.11.2016. no: 70198063-050.06.04 16-10/), adult patients who applied to our clinic with distal tibial metaphyseal fractures, were treated with plate osteosynthesis, and had at least 12 months of follow-up were included in the study. The study was conducted in line with the principles of the Helsinki Declaration. According to the Arbeitsgemeinschaft für Osteosynthesefragen (AO) classification, patients were classified as having fractures of A, B, or C types. Cases of Gustilo-Anderson Type III open fractures, severely damaged extremity injuries, pathological fractures, and age <18 years were excluded, as were patients who could not be followed after treatment.

Surgical technique

**Figure 1:**
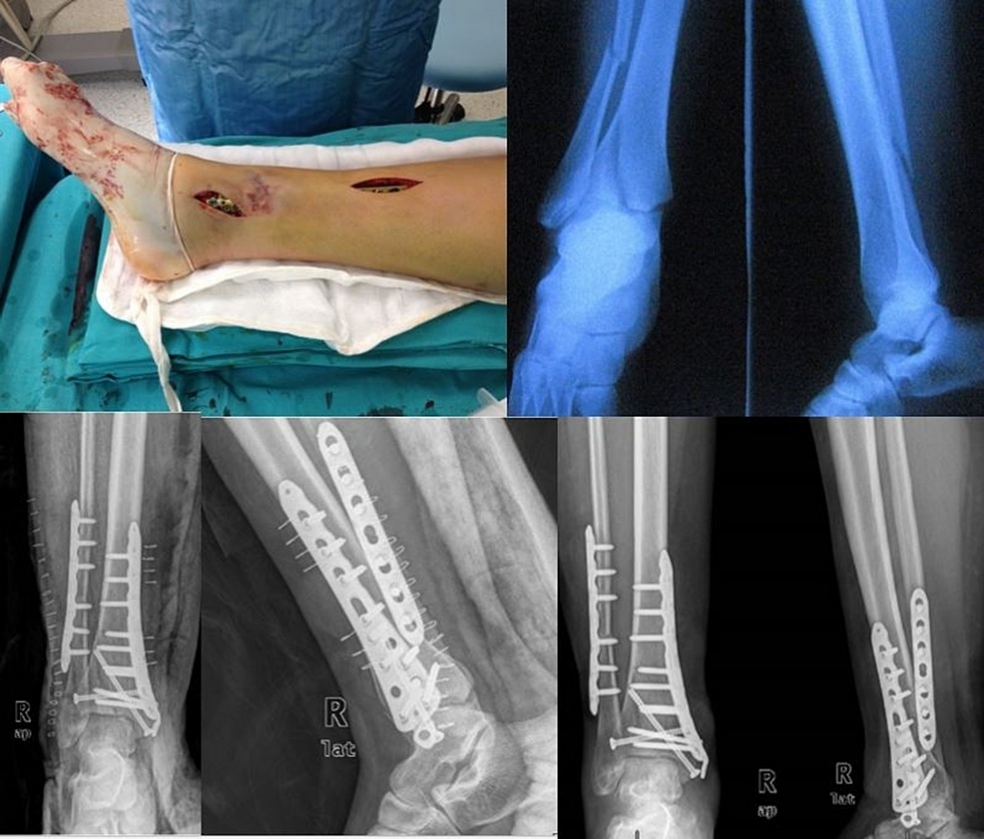
Minimally invasive plating Distal medial anatomical plate placement with a minimally invasive technique. Preoperative and early postoperative X-rays. Cannulated screws were used for joint surface restoration. Second-year control after the operation.

**Figure 2:**
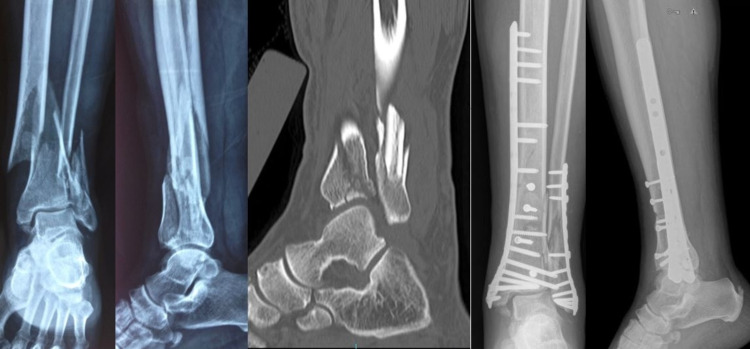
Open technique Treatment of a severely displaced distal tibial intra-articular fracture with open reduction and internal fixation

Fracture reduction was performed under fluoroscopy following general/spinal anesthesia in the application of the minimally invasive surgical technique. Joint dehiscence was addressed with the use of percutaneous cannulated screws. General fixation of the fracture was achieved with a distal tibia medial anatomical plate and screws that slide over the periosteum through the incision made over the medial malleolus. Distal screws were applied with an incision into the medial malleolus, and proximal screws were applied via a single mini-incision (Figure 1). For patients who underwent open reduction, the fracture site was reached with a medial or anterior incision appropriate to the specific case. Intra-articular fragments and other fracture fragments were reduced openly and the distal tibia was fixed with a medial or lateral anatomical plate (Figure 2). For both techniques, in the case of a lateral malleolar fracture, the fibula was fixed with open reduction and internal fixation or K-wire.

After the surgical procedure, patients in both groups were followed with the same protocol, which included a short leg splint for two weeks postoperatively until soft tissue healing was completed. After the sutures were removed, ankle movements were begun together with partial load mobilization. After the first month, 50% weight mobilization was begun, reaching 100% after the second month. Low-molecular-weight heparin and acetylsalicylic acid were given for the first 10 days and then for one month as thromboprophylaxis. As antibiotic prophylaxis, first-generation cephalosporin was administered intravenously for 48 hours. In the event of incomplete wound healing or deep infections, cultures were taken from wound areas and antibiotic therapy was continued according to the results.

Patient data were obtained from the hospital automation system and entered into Microsoft Excel (Microsoft Corporation, Redmond, WA). Age, gender, side, fracture type, and plating type (open reduction or minimally invasive) data were recorded. The anterior distal tibial angle (ADTA) and lateral distal tibial angle (LDTA) were calculated from films obtained in control visits with digital radiological data via the hospital’s automation system [9]. Clinical evaluation was performed according to the American Orthopaedic Foot and Ankle Society Ankle-Hindfoot Score (AOFAS) [10]. 

In subsequent radiographs, bridging and callus formation at the bone ends in at least three cortices were evaluated as union. Nonunion was defined as osteolysis at the fracture ends, absence of blunting of the fracture ends, and implant failure in the following radiographs. Skin necroses that healed with dressing were considered superficial. Deep infections were infections that required debridement or removal of the implant in the operating room.

Patients were divided into two groups, those who underwent minimally invasive or those who underwent open surgery. Clinical and radiological outcomes were compared between both groups. Additional comparative analysis was performed for each fracture type (A, B, C) for each surgical method.

Statistical analysis

IBM SPSS Statistics 25 (IBM Corp., Armonk, NY) was used for statistical evaluation. Means and standard deviations were used in the evaluation of numerical data. Percentages were used in the evaluation of proportional data. The Shapiro-Wilk test was applied to determine whether the means of numerical data among groups had a normal distribution. Crosstables and chi-square tests were used to evaluate categorical data. Fisher-exact tests were used when the value obtained in the table for categorical data was less than 5. Statistical significance was accepted as 0.05 within 95% confidence intervals.

## Results

A total of 44 patients with adequate follow-up from among 53 patients were included in this study. The mean follow-up period was 20.73 (12-50) months. Twenty patients were male and 24 were female. The mean age of the patients was 44.98±16.48 (18-75) years. While 27 patients presented due to falls, 17 patients had suffered high-energy trauma (traffic accidents, falling from heights, etc.). The fracture classifications of the patients according to the AOFAS included 13 cases of A, 14 cases of B, and 17 cases of C-type fractures. Three of the patients had open fractures that could be regarded as type 1. The fibula was intact in seven cases, and in the cases of four patients, it was fractured, but no fixation was performed (shaft or proximal). Fibula fixation was performed for 33 patients (29 plates, 4 K-wires) (Table 1).

**Table 1:** Demographics General patient distribution (*) Mann Whitney U test. (**) Pearson chi-square test (***): Fisher's exact test L.E.: low-energy, H.E.: high-energy

				Minimal invasive			Open Reduction			p-value
Age				42.25 +/- 18.017			47.25 +/- 15.092			0.126*
Gender			Male	7	35%		13	54.2%		0.204**
			Female	13	65%		11	45.8%		
Side			Right	11	55%		9	37.5%		0.246**
			Left	9	45%		15	62.5%		
Fracture type (AO)			A	7	35%		6	25%		0.234**
			B	8	40%		6	25%		
			C	5	25%		12	50%		
Follow up (months)				18.40 +/- 8.319			22.67 +/- 10.453			0.189*
Fracture mechanism		L.E.		13		65%	14		58.3%	0.651**
		H.E.		7		35%	10		41.7%	
Open fracture				2		10%	1		4.2%	1.000 ***
Fibula	Fixed			14		70%	19		79.2%	0.484 **
	İntact / no fixation			6		30%	5		20.8%	

Considering the division of the patients into two groups as those treated with minimally invasive or open reduction methods, 20 patients were treated with the minimally invasive approach and 24 patients were treated with the open reduction method. In the radiological results of patients treated with the minimally invasive method, the mean ADTA was 92.85 (90-105) degrees, and the mean LDTA was 82.55 (78-95) degrees. The mean union time was 4.37 (3-8) months. There was nonunion in only one case (5.0%). Clinically, the mean AOFAS was 92.15 (61-100). Among the radiological results of the patients treated with the open reduction method, the mean ADTA was 91.04 (80-100) degrees and the mean LDTA was 80.71 (75-90) degrees. The mean union time was 4.17 (3-10) months. There was nonunion in only one case (4.2%). Clinically, the mean AOFAS was 90.04 (60-100). There were no statistically significant differences in ADTA, LDTA, union time, or AOFAS between the two groups (p>0.05) (Table 2).

**Table 2:** Clinic and radiologic results Comparison of clinical and radiological data between the two groups (*) Mann Whitney U test. (**) Fisher's exact test. ADTA: anterior distal tibial angle. LDTA: lateral distal tibial angle. AOFAS: American Orthopaedic Foot and Ankle Society Ankle-Hindfoot Score.

	Minimal invasive		Open reduction		p value
	mean		mean		
ADTA	92.85	+/- 4.308	91.04	+/- 4.832	0.314*
LDTA	82.55	+/- 5.145	80.71	+/- 3.884	0.964*
AOFAS	92.15	+/- 7.916	90.04	+/- 8.073	0.209*
Union time (months)	4.37	+/- 4.368	4.17	+/- 4.174	0.258*
Union rate (%)	95.0		95.8		1.000**

When comparing the clinical scores, complications, and union rates among the fracture types between two groups, complications and union rates were similar in all fracture types (p>0.05). Similar clinical outcomes regarding AOFAS were obtained in patients with type A and B fractures (p>0.05). Patients with type C fractures seemed to have statistically better clinical outcomes in the minimally invasive surgery groups (p=0.015).

When complications were compared between the two groups, one case of nonunion was identified in each group. Superficial skin problems were seen in four (20%) patients in the minimally invasive group and in eight (33.3%) patients who underwent open reduction. Deep infections were seen in three (15%) patients in the minimally invasive group and in two (8.3%) patients in the open reduction group. Implant removal was performed due to irritation for three (15%) patients in the minimally invasive group and four (16.67%) patients in the open reduction group. When the complication rates were compared, there was no statistically significant difference (p>0.05) (Table 3).

**Table 3:** Complications List of complications (*): Fisher's exact test. (**): Pearson chi-square test

	Minimal invasive		Open reduction		p-value
Nonunion	1	5%	1	4.2%	1.000*
Superficial skin problems	4	20%	8	33.3%	0.323**
Deep infection	3	15%	2	8.3%	0.646*
Implant removal	3	15%	4	16.7%	1.000*

## Discussion

The soft tissue coverage of the distal tibial region is limited and the blood supply is weak. Therefore, infections and skin problems are common. After fractures of this region, soft tissue problems and infections are seen at a general rate of 6-37% [11]. In order to overcome this problem, minimally invasive methods are being developed to avoid damage to the soft tissues with additional dissection in surgery [12]. 

The most important problems encountered in the treatment of distal tibial fractures with open reduction are superficial skin necrosis and deep infections [11,13]. However, an anatomical reduction can be achieved through open reduction, which is very important for long-term results [3]. The rate of deep infection in our patients who underwent open reduction was 8.3% (2 patients), similar to the rates reported in the literature. The mean AOFAS of our patients who underwent open reduction was 90.04 (“good to excellent”).

The most important expected advantages of the minimally invasive method are less skin necrosis, less deep infection, and better union. In previous studies, deep infection was reported at rates between 0% and 14% with the minimally invasive technique [6,9,14-15]. Because the plate does not fully adhere to the bone, skin necrosis, deep infection, and plate removal may also occur with the minimally invasive method [15]. There were four (20%) cases of skin problems, three (15%) cases of deep infection, and three (15%) implant removals due to irritation in the patient group that we treated with a minimally invasive approach. Thus, it was seen that the minimally invasive method could not completely resolve soft tissue problems and deep infections.

Previous studies have compared minimally invasive and open reduction in the treatment of distal tibial fractures [16-20]. In these comparative studies, less skin necrosis and fewer deep infections were reported among the minimally invasive groups. Our patient group showed similar results. Clinically, according to AOFAS values, Kim et al. reported an average of 86 points in the minimally invasive group and again 86 in the plate-treated group. Thus, there was no statistical difference between the two groups [16]. Gülabi et al. reported similar results (78.7-78.8, p>0.05) [20]. In our patient group, the mean AOFAS score was 92.15 in the minimally invasive group and 95.8 in the open reduction group. There was no statistically significant difference between the two groups (p>0.05). In their angular evaluation, Kim et al. found more coronal angulation in their minimally invasive group [16]. There was no such finding in our patient group, and there was no statistically significant difference between the ADTA values of these groups. When compared between different fracture types we observed that only patients with type C fractures had a significant benefit from minimally invasive surgery.

The application of minimally invasive plate osteosynthesis (MIPO) is a good method for avoiding problems with soft tissue. However, the entry point is above the medial malleolar in these cases. This area is where soft tissue problems are most likely to arise. In addition, soft tissue problems can be seen with the MIPO method due to plate irritation and skin nutrition deterioration if the plate remains exposed to air while being positioned via the small incision site. Zou et al. found plate irritation in nine patients treated with a minimally invasive method and in two patients treated with open reduction. They reported that the minimally invasive method was not very advantageous [19]. In our study, implant removal was performed for three patients treated by the minimally invasive method and four patients in the open reduction group due to plate irritation.

Since open reduction requires soft tissue dissection, it carries a risk of skin necrosis and deep infection. However, in cases where the joint is severely displaced, metaphyseal crush fractures must be considered for patients who require a graft due to the defect. Among our cases, superficial infections developed in eight patients who underwent open reduction, while deep infections developed in two patients. In the minimally invasive group, we found four superficial skin problems and three deep infections. Although the infection rates were similar, the rate of skin necrosis was slightly higher in the open reduction group.

There are some limitations of our study; The most important of these is the small number of our patients. In addition, randomization could not be done because we have a retrospective study design. Therefore, we did not have the chance to select patients with similar demographic characteristics in the comparison of the two groups. The distal tibia region has many variables due to its structure. There are many variables that can affect the clinical and radiological outcome such as the type of fracture, fracture mechanism, the condition of the fibula, and soft tissue coverage. These reasons make an exact peer comparison between the two groups very difficult.

## Conclusions

MIPO and open surgery can be chosen in the treatment of distal tibial fractures with satisfying results. Although both techniques are effective and have similar clinical outcomes, MIPO seems to be advisable among patients with type C fractures. During MIPO application, attention should be paid to the soft tissues. Soft tissue problems can still be seen even with the application of a minimally invasive method.
